# Diagnostic tests for Crimean-Congo haemorrhagic fever: a widespread tickborne disease

**DOI:** 10.1136/bmjgh-2018-001114

**Published:** 2019-02-20

**Authors:** Laura T Mazzola, Cassandra Kelly-Cirino

**Affiliations:** Emerging Threats Programme, Foundation for Innovative New Diagnostics (FIND), Geneva, Switzerland

**Keywords:** crimean-congo haemorrhagic fever, CCHF, in vitro diagnostics, outbreak

## Abstract

Crimean-Congo haemorrhagic fever (CCHF) is a widespread tickborne disease that circulates in wild and domestic animal hosts, and causes severe and often fatal haemorrhagic fever in infected humans. Due to the lack of treatment options or vaccines, and a high fatality rate, CCHF virus (CCHFV) is considered a high-priority pathogen according to the WHO R&D Blueprint. Several commercial reverse transcriptase PCR (RT-PCR) and serological diagnostic assays for CCHFV are already available, including febrile agent panels to distinguish CCHFV from other viral haemorrhagic fever agents; however, the majority of international laboratories use inhouse assays. As CCHFV has numerous amplifying animal hosts, a cross-sectoral ‘One Health’ approach to outbreak prevention is recommended to enhance notifications and enable early warning for genetic and epidemiological shifts in the human, animal and tick populations. However, a lack of guidance for surveillance in animals, harmonisation of case identification and validated serodiagnostic kits for animal testing hinders efforts to strengthen surveillance systems. Additionally, as RT-PCR tests tend to be lineage-specific for regional circulating strains, there is a need for pan-lineage sensitive diagnostics. Adaptation of existing tests to point-of-care molecular diagnostic platforms that can be implemented in clinic or field-based settings would be of value given the potential for CCHFV outbreaks in remote or low-resource areas. Finally, improved access to clinical specimens for validation of diagnostics would help to accelerate development of new tests. These gaps should be addressed by updated target product profiles for CCHFV diagnostics.

**Summary box**

- Diagnostic tests for Crimean-Congo haemorrhagic fever virus (CCHFV), a WHO R&D Blueprint priority pathogen, include commercial reverse transcriptase PCR and serological diagnostic assays and multiplex panels to distinguish from other viral haemorrhagic fever agents.
- Despite the extensive range of tests available, diagnostic gaps remain, including a need for improved surveillance for early detection, a lack of point-of-care testing options and issues with limited availability of clinical specimens for test validation.
- Refinement of target product profiles for CCHFV diagnostics to include these needs will help to enhance surveillance, prevention and management of CCHFV in both human and animal hosts.

## Introduction

Crimean-Congo haemorrhagic fever (CCHF) is one of the high-priority pathogens identified on the WHO R&D Blueprint because of its high case fatality rate, potential for nosocomial outbreaks and difficulties in treatment and prevention.^1–3^ CCHF is widespread, now found in Europe, Asia, Africa, the Middle East and the Indian subcontinent, with currently no vaccine available for widespread human or animal use. This landscape analysis is intended to provide a view to the current state of CCHF diagnostics, with particular emphasis on human diagnostics for screening, diagnosis and surveillance.

### History and epidemiology of CCHF

**Figure 1:**
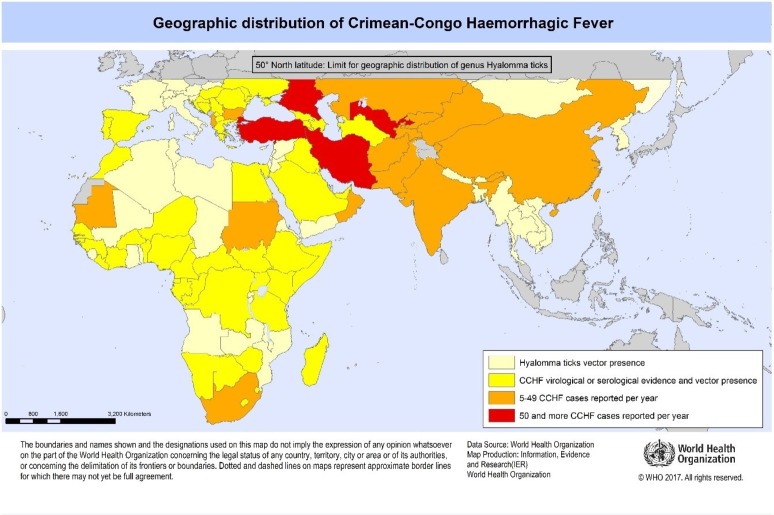
Geographical distribution of Crimean-Congo haemorrhagic fever (CCHF) (http://www.who.int/emergencies/diseases/crimean-congo-haemorrhagic-fever/en/).

CCHF virus (CCHFV) is an orthonairovirus of the family Nairoviridae causing severe and often fatal haemorrhagic fever in humans. The disease was first described in the Crimea in 1944 and became known as ‘Crimean haemorrhagic fever’, but the virus was first isolated in Congo in 1956 and was named ‘Congo virus’; the two names converged as CCHF in 1969.^2 4 5^ CCHF is considered an ‘emerging’ disease across the globe, with many countries reporting new infections in recent decades.^6–8^ CCHF is now found in Europe, the Mediterranean, China and Central Asia, Africa, the Middle East and India (figure 1), and has been reported after long periods of absence.^9 10^ Cases are typically sporadic and seasonal, and occur in remote or agricultural regions.

### CCHF reservoir and mode of transmission

Ixodid (hard) ticks are a reservoir and a vector for CCHFV, now commonly found in dry or semiarid environments across Africa, Europe, the Middle East and Asia.^7 11 12^ Numerous wild and domestic animals, including cattle, goats, sheep, camels and hares, serve as amplifying hosts after being bitten by infected ticks, but animal infections are difficult to detect as the tick–animal–tick cycle is asymptomatic.^13^ CCHFV persists throughout the tick life-cycle, enabling reservoirs of infection over long periods without vertebrate hosts.

Transmission to humans occurs most frequently among agricultural workers following the bite of an infected tick, and among slaughterhouse or veterinary workers exposed to the blood and tissue of infected livestock. Human-to-human transmission is infrequent, but has arisen from contact with infected patients or contaminated medical equipment.^14–17^

### Clinical presentation

CCHFV infection typically has four distinct phases—incubation, prehaemorrhagic, haemorrhagic and convalescence. The incubation period is in the range of 1–5 days following a tick bite, and 5–7 days following contact with infected blood or tissues.^4 11 16^ The prehaemorrhagic phase is characterised by the sudden onset of a wide range of non-specific symptoms that mimic other diseases and lasts for 4–5 days. CCHFV infections may be subclinical or asymptomatic in some people; for example, high levels of seroprevalence have been detected in specific regions of Turkey and Greece.^18 19^

The haemorrhagic phase is generally 2 weeks in duration with rapid progressive haemorrhage. Severely ill patients may experience rapid deterioration, shock and multiorgan failure.^20^ In documented outbreaks, fatality rates in hospitalised patients have ranged as high as 50%, with an average mortality of 30% occurring in the second week of illness.^1 2^ For survivors, recovery generally begins 10–20 days after the onset of illness; however, full recovery may take up to a year.^11 16^ The long-term effects of CCHFV infection have not been studied well enough in survivors to determine whether or not specific complications exist.

### Molecular epidemiology

CCHFV displays a high degree of sequence diversity, with divergence of 20%, 31% and 22% for the S, M and L segments among isolates in GenBank. Based on an analysis of the viral S segment, six or seven viral lineages/clades have been identified,^6 11 16 21 22^ suggesting a lengthy history of geographical dispersion of the virus from Africa to Europe, the Middle East and then Asia. Genome diversity can be greater in the tick than in the mammalian host.^23–25^ The extensive sequence diversity of CCHFV is likely due to genetic reassortment, enhanced by circulation and adaptation of strains into new geographical regions.^22 26–29^

### Therapeutic efforts

Treatment for confirmed cases of CCHF is general supportive care with management of symptoms.^2^ There is no approved antiviral treatment. Although the antiviral drug ribavirin is widely used based on its in vitro activity, clinical evidence is inconclusive.^30–36^ Postexposure prophylaxis using ribavirin is controversial but recommended as a safe option for healthcare providers with high-risk/direct exposure to CCHF-infected patient fluids or tissue.^15 17 37 38^

Development of new therapies has been slow in part due to the requirement for a high-containment laboratory and the lack of a suitable animal model.^6 39 40^ Favipiravir is a broad-spectrum RNA inhibitor that has shown promising results.^41^ Tetracyclines, cationic amphiphilic drugs and ubiquitin variants are under investigation as potential antiviral therapies^42–44^; however, none are in clinical-stage development.

### Vaccine efforts

There is currently no safe and effective CCHF vaccine widely available for human use.^1 2^ A vaccine developed in 1970 was based on CCHFV cultivated in suckling mouse brain. Vaccinated individuals developed anti-CCHFV antibodies, but the neutralising activity was low.^45^ The vaccine is licensed in Bulgaria and used on a small scale in eastern Europe, but it is unlikely to gain international regulatory approval due to concerns with efficacy and allergic responses.^46^ Several studies indicate that neutralising antibodies alone are insufficient protection against CCHFV challenge,^39 47 48^ and it is unclear whether conserved neutralising epitopes are present for all CCHFV isolates.^49^

Newly developed mouse models that mimic human CCHF disease are useful to study vaccine candidates. A recent study vaccinated mice with a DNA vaccine (encoding a ubiquitin-linked version of CCHFV Gc, Gn and NP) and demonstrated 100% efficient preventive immunity against lethal CCHFV challenge.^39^ Using this mouse model, vaccination with a cell culture-based vaccine from the CCHFV Turkey-Kelkit06 strain provided partial protection (80%) against a high-dose challenge.^47^ A recombinant vaccine expressing CCHFV glycoproteins (GP, M segment) using a modified vaccinia virus Ankara (MVA) protected 100% of recipient animals up to 14 days postchallenge in a lethal challenge model adapted to represent infection via a tick bite.^50^ However, a similar study using MVA to express the CCHFV nucleoprotein (NP, S segment) generated an immune response but failed to protect animals from lethal disease.^51^

Veterinary vaccines may be an alternative or complementary approach to human vaccines. Livestock vaccines against CCHF could play an important role in preventing human infection by controlling exposure during animal slaughter, as well as interrupting the vector cycle during tick feeding.^8 46^ Infection control of CCHFV in wildlife populations will remain a challenge, however. Currently there are no vaccines available for use in animals,^1 2^ although the MVA GP vaccine is currently being evaluated in sheep.^52^

## CCHF diagnostics

Laboratory tests used to diagnose CCHF include reverse transcriptase (RT)-PCR, immunofluorescence assay (IFA), antibody (IgG, IgM) and antigen-capture ELISA, and virus isolation. Patients suspected of CCHF are primarily diagnosed by RT-PCR as these assays provide the highest detection sensitivity to active infection at the earliest time point. Lineage detection may be challenged by the high diversity and in situ evolution of CCHFV, particularly for RT-PCR assays which rely on a conserved genomic sequence for detection.^53–57^ Serological detection is less impacted by minor genomic variations. Given CCHFV strain variations, it is recommended that nucleic acid amplification tests (NAAT, eg, RT-PCR) be used in combination with immunological assays for highest detection sensitivity;^8 58 59^ however many low-resource settings may not have the capacity for PCR testing, especially at the early stages of an outbreak. Virus isolation is rarely used as a diagnostic tool because of the stringent biosafety containment level (BSL-4) required.

NAAT typically requires the highest laboratory infrastructure, including biosafety hoods and a clean room or PCR workstation, while most serological tests (ELISA, IFA) can be run on the benchtop in a more modest laboratory environment (table 1).^60 61^ Ideally, point-of-care (POC) NAAT tests are fully automated, with samples delivered to an integrated cartridge that contains all the reagents necessary for sample processing and analysis; this process can be performed without a biosafety hood, depending on the sample preparation requirements (here defined as BSL-2 for human disease).^60 61^ Once the cartridge is inserted into the instrument, no further manual steps are required. Rapid diagnostic tests (RDTs) are typically designed for field or home use. Turnaround time for each test is specified by the manufacturer; turnaround time per result can include additional time (days to weeks) for sample transport and processing at the reference lab.^62–64^

**Table 1:** Diagnostics infrastructure comparison

Test type	Infrastructure requirements	Training requirements	Turnaround time	Inhouse or prototype	Commercial source	Target population
Virus isolation, neutralisation	High (BSL-4) (reference laboratory)	High (advanced lab technician)	3–7 days	Several	–	Human, animal
NAAT reference (including multiplex)	High (BSL-3/4) (regional lab, reference laboratory)	High to moderate (advanced lab technician)	2.5 hours 1–2 hours prep	>10	>5	Human, animal, ticks
NAAT POC	Moderate/BSL-2 (district hospital)	Moderate (laboratory technician)	1–2 hours	1	–	Human, ticks, culture
ELISA, IFA	High to moderate (regional lab, district hospital)	Moderate (laboratory technician)	3–4 hours	>10	6	Human, animal, culture
RDTs	Low (clinic, health centre, field settings)	Low (nurse, healthcare worker)	<30 min	–	–	–

BSL, biosafety containment level; IFA, immunofluorescence assay; NAAT, nucleic acid amplification test; POC, point of care; RDT, rapid diagnostic test.

Several commercial assays for PCR and serology are available (online supplementary table S1 and S2), although the majority of international laboratories use inhouse assays, likely due to an investment in tests developed from regional CCHV strains.^7 8 53 59^ It has also been suggested that commercial tests may be too expensive, difficult to order or not available to international customers.^8 65^ The majority of the inhouse assays have a publication history, several with published data on diagnostic performance or external quality assessment (tables 2 and 3).

10.1136/bmjgh-2018-001114.supp1 Supplementary data

**Table 2:** Inhouse NAAT tests for CCHF: references, reviews, EQA

Test type	Reference	Review/EQA	Labs using the method (n)^65^	CCHFV target^54^
qRT-PCR	73	54 65	3	24 genomic targets
qRT-PCR	113	54	–	19 strains worldwide
qRT-PCR	76 78	53 54 59 65	2	Kosovo Hoti and Drosdov strains
qRT-PCR	114, updated 57	54	–	18 strains worldwide (updated 26)
qRT-PCR	115 116	65	3	–
qRT-PCR	117 118	53 54 59 65	12	17 strains worldwide
qRT-PCR	119	53 59 65	2	–
qRT-PCR	120	54	–	19 Southern African strains
qRT-PCR	121	54	–	All known worldwide strains, including the AP92 strain
qRT-PCR	122	–	–	–
Nested RT-PCR	123	53 59 65	2	–
Nested RT-PCR	74	53 65	2	–
Nested RT-PCR	22	65	1	–
Nested RT-PCR	124	–	–	–
Conventional RT-PCR	72 125	54 65	2	7 geographically diverse strains
Isothermal RPA	82	–	–	7 geographically diverse strains
PCR multiplex	64 92	–	2	–
Low density array	118	53 54	–	Strains worldwide
High density array	90	54	–	Nigerian strain
Multiplex RT-PCR/NGS	84	–	–	46 VHF species

CCHF, Crimean-Congo haemorrhagic fever; CCHFV, CCHF virus; EQA, external quality assessment; NAAT, nucleic acid amplification test; NGS, next-generation sequencing; qRT-PCR, quantitative real-time RT-PCR; RPA, recombinase polymerase amplification; VHF, viral haemorrhagic fever.

**Table 3:** Inhouse serological tests for CCHF: references, reviews, EQA

Test type	Reference source	Review/EQA
ELISA (Ag, IgG, IgM)	87 126	8
ELISA (IgG, Ag)	104 127	53/8
ELISA (IgG)	128	8
ELISA (IgG, IgM)	129	8
ELISA (IgG, IgM)	130	53/8
ELISA (IgG)	131	53/16
ELISA (IgG)	132	8 16
ELISA (IgM)	133	8
ELISA (IgG)	134	–
Competitive ELISA	134	
Double-Ab ELISA	100	–
Immune complex ELISA	135	–
Multiplex ELISA	91	(7 viral species)
IFA (IgG, IgM)	87	8
IFA (IgG)	136	53/8
IFA (IgG)	137	8

CCHF, Crimean-Congo haemorrhagic fever; EQA, external quality assessment; IFA, immunofluorescence assay.

### Specimens and sampling

CCHFV is classified as BSL-4 in some countries, but if serum samples have been inactivated (eg, with virucides, gamma rays, formaldehyde, heat and so on), they can be manipulated in a basic biosafety environment.^1 2^ Otherwise, CCHF patient samples present an extreme biohazard risk and should only be conducted under maximum biological containment conditions.^8 11 15^

CCHFV can be detected in the saliva and urine from prehaemorrhagic stage patients, consistent with other viral haemorrhagic fevers (VHF).^66–69^ CCHFV has also been detected in nasal, conjunctival, rectal and vaginal swabs of severe cases.^70^ A more thorough study of CCHF viral kinetics is needed to determine whether viral RNA peaks in serum during early-stage infection, then later in urine during the recovery stage as with other VHF.^71^ In particular, urine and saliva samples may be desirable for easy collection and handling for CCHF diagnosis. Culture of CCHFV from urine indicates the potential for prolonged viraemia up to 30 days, as well as potential transmission risk in recovering patients.^34 37^

### Molecular diagnostics

RT-PCR-based techniques typically target the nucleoprotein gene region in the S segment, which is the more conserved region of the CCHFV genome across geographical isolates.^72–75^ CCHFV RNA peaks in the first week after symptom onset and can be detected for up to 3 weeks.^58 72 75 76^ Viral load, which varies considerably among patients with CCHF, can be an indicator of severity.^73 77–80^ For moderate cases, serum viral loads are initially 10^2^–10^4^ copies/mL, while severe cases have initial viral loads typically 10^4^–10^7^ copies/mL, with 10^8^–10^10^ copies/mL predictive of fatal outcome.^77 78 81^

Quantitative real-time RT-PCR (qRT-PCR) has better performance over conventional RT-PCR or nested RT-PCR, with a lower contamination rate, higher sensitivity and specificity, and better time-effectiveness^59 65^; laboratories performing only nested or conventional RT-PCR have been advised to implement qRT-PCR to improve assay performance and viral load determination. An isothermal NAAT test has been developed based on recombinase polymerase amplification, enabling amplification at a single temperature in a more ‘crude’ sample which may prove more amenable as a field diagnostic or in low-resource laboratories.^82^ Next-generation sequencing (NGS) enables comprehensive genome analysis and has been used for CCHFV phylogeny^83 84^; however, this complex and expensive approach is not currently practical for diagnostic screening.

There are many inhouse laboratory tests, some with primers designed for regional circulating strains (table 2).^53 59 85 86^ Several commercial RT-PCR kits are available, typically with primers designed to target broad consensus sequences within the S segment, including several European Commission marked (CE) diagnostics and research use-only (RUO)-labelled products in stand-alone and multiplex test formats (online supplementary table S1).

### Serological assays

Serological assays are sensitive to antigenic variation, but generally less impacted by genetic variation.^53^ Most assays target the CCHFV N protein, which induces an early, strong and long-lasting immune response in humans.^86^ Active CCHFV infection can be detected by IgM or a significant increase in IgG titre following the acute phase of infection 4–9 days after symptom onset; however, severe and fatal cases often do not mount a detectable antibody response.^79 87 88^ Detection of anti-CCHFV IgG can indicate current or resolved infection (often years after infection) and can be useful in surveillance epidemiological studies. For CCHFV, capture ELISA has been shown to be more sensitive than IFA or neutralisation assay.^87–89^ Virus neutralisation assays are less useful for diagnosis, since CCHFV elicits relatively low levels of neutralising antibodies, but can be useful for epidemiology and vaccine research. CCHFV neutralisation is generally performed using plaque reduction neutralisation, with 5–7 days for results.

As with RT-PCR, many of the CCHFV serological tests in use were developed as inhouse assays with limited validation (table 3).^8 53 59^ Several commercial ELISA (IgG, IgM) and IFA kits are available, although primarily marketed as RUO (online supplementary table S2).

10.1136/bmjgh-2018-001114.supp3 Supplementary data

### Rapid diagnostic tests

RDTs can leverage the same antibody/antigen capture agents as an ELISA but in a lateral flow strip format, with minimal specimen processing (blood, plasma, swabs). This enables a faster time to result (10–30 min, however with a lower detection sensitivity than ELISA, due in part to reduced sample volume).^90^ RDTs are ideal screening tests, suitable for field testing and low infrastructure settings,^87^ although follow-up confirmatory testing is often required. RDTs have been used to effectively screen and triage suspected high-risk cases of diseases such as Ebola and dengue^88 89^; however, the literature shows no evidence of CCHF RDT development. The primary challenge is detection sensitivity, as the IgG/IgM serological response is typically detectable only 5 days postinfection, and often undetectable in severe and fatal infections.

### Syndromic multiplex approach

CCHF screening tests need to be able to distinguish CCHF from other types of VHF, particularly in regions where VHF viruses may be endemic and maintained in the region in natural reservoirs. In some instances, a multiplex approach may be the better option for definitive identification, or at least to rule in or rule out more virulent pathogens.^4 7 58^

For analysis of circulating reservoirs in Sierra Leone, a bead-based immunoassay was used to detect IgG antibodies for multiple pathogens, which included Lassa, Ebola, Marburg and Rift Valley fever viruses, CCHFV, and pan-assays for flaviviruses and alphaviruses.^91^ In 675 human serum samples, 50% were positive for Lassa, 5% for Ebola, 11% for Marburg, 2% for Rift Valley fever, 2% for CCHF, 53% for flaviviruses and 56% for alphaviruses. A multiplex PCR approach developed as a universal array for simultaneous identification of Ebola, Marburg, CCHF, Lassa fever, Rift Valley fever, dengue and Yellow fever, as well as *Variola* and *Vaccinia* virus for smallpox, detected all viruses in 32 different isolates, with no cross-reactivity with other emerging viruses.^64^ Finally, a qRT-PCR-based card-based platform developed for 26 acute febrile illnesses,^92^ including 15 viruses, 8 bacteria and 3 protozoa, achieved an overall 88% sensitivity and 99% specificity compared with individual real-time RT-PCR assays.^73^

In addition, febrile agent panels (20 and 10 member panels including CCHF) are recently commercially available using bead-based and real-time TaqMan assays with a limit of detection of 10 copies/mL (online supplementary table S1).

## Challenges for CCHF diagnostics

### Surveillance

Surveillance programmes for humans, animals and ticks in endemic and bordering non-endemic areas can be used to monitor the spread of disease.^8 93^ As infected animals are usually asymptomatic, only active surveillance or human case detection will reveal CCHFV in circulation. Seroconversion in animals is a good indicator of CCHFV prevalence; when domestic animals in Turkey and Bulgaria were tested for CCHFV-specific IgG antibodies, the mean seroprevalence was 26% for Bulgaria and 57% for Turkey, with some provinces reporting seroprevalence of almost 90%.^94^ In both rural and urban settings, similar ‘random sampling’ surveillance programmes have been employed for ticks^85 95–97^ and other ruminants.^98 99^ However, routine reservoir/host monitoring is not broadly implemented, and surveillance is challenged by a lack of serodiagnostic tests suitable for large-scale animal testing,^100^ no clear guidance for standardised surveillance of CCHFV in the animal health sector, and the cost of routine implementation.^6^ For human surveillance, high prevalence endemic countries (Iran, Iraq, Pakistan and Turkey) report human cases annually through health surveillance systems, although not uniformly effective.^101^ Other countries (Afghanistan, Egypt, Oman, Saudi Arabia and United Arab Emirates) have occasional human cases reported; these and surrounding non-endemic countries would benefit from active surveillance systems for early identification of hot spots.^102^

### Harmonisation of case definition

For CCHF surveillance, harmonisation of case identification is necessary to enhance notifications and estimate disease burden, as well as to enable early warning for genetic and epidemiological shifts in the human, animal and tick populations.^6 59 95 103^ National CCHF prevention and control programmes should be strengthened and supported by the respective Ministries of Health and international agencies.^6 63 102^ To assist these goals, a guideline development group for CCHF has been established by WHO to formulate recommendations, evaluate optimal implementation and develop guidelines on clinical management,^104^ as well as ongoing efforts towards the WHO Roadmap to prioritise research and product development for CCHF.^105^

### Clinical validation

During the early stage of an outbreak, diagnostic tests are often evaluated using the strains most relevant to that region. Diagnostic test development could be accelerated through validation and external quality control (EQA) using up-to-date clinical specimen panels and reference standards, particularly since prior EQA performance indicated a wide range in laboratory test sensitivity. While the majority of laboratories received high marks, the observed sensitivities ranged from 75% to 100% for serological assays and from 43% to 100% for molecular assays (with outliers as low as 25% for older test methods).^53 65^ Specifically, routine EQA studies should include a range of CCHFV genotypes and concentrations to accurately evaluate and compare diagnostic performance. To the extent possible, patient specimens could be characterised and maintained for diagnostic test evaluation and quality assurance. In the absence of clinical specimens, a recombinant approach may be needed to generate sufficient quantities of quality control material.^106 107^

For CCHF diagnostic test developers, sourcing clinical specimens has been a major roadblock to both molecular and serological assay validation.^8^ The manufacturing process requires a substantial amount of reference material, and often companies develop inhouse calibration standards to control supply and lot-to-lot variability. There is little incentive to seek international regulatory approval; even for commercial suppliers, the investment for regulatory approval is often subject to market demand. Some international reference institutes, including the WHO International Biological Reference custodian laboratories, provide reference materials or specimen panels for validation or EQA/proficiency (online supplementary list S1). A specimen bank would be beneficial to CCHFV diagnostic development; however, this effort faces significant challenges given the sporadic nature of human cases which typically occur in remote agricultural regions across 30 countries, with only several hundred cases confirmed each year.^6^

10.1136/bmjgh-2018-001114.supp2 Supplementary data

### Turnaround time and POC testing

As RT-PCR testing requires a high infrastructure laboratory and a turnaround time of 2–5 days, a more flexible approach is needed for an outbreak, with options that serve both animal and human populations. POC and ‘near-POC’ molecular diagnostic platforms have significantly lower infrastructure requirements and have been implemented in decentralised laboratories.^108–110^ These POC instruments are compact and self-contained, with automated sample preparation, and most healthcare workers can be trained for operation in clinic or field-based settings. Given the range of assays already developed for these commercial platforms, it is likely that current CCHFV RT-PCR assays could be readily adapted to the POC cartridge-based format.

## Conclusion

This analysis identified several commercial sources for CCHF molecular diagnostics and serology, as well as a large number of inhouse tests. Despite this, several of the gaps identified in the 2016 WHO R&D Blueprint remain.^3 111^ A more detailed understanding of CCHF viral and antibody kinetics is needed across the broad range of sample types. Routine EQA, using well-characterised and up-to-date specimen panels, would be valuable for both clinical validation and proficiency testing. Surveillance is currently limited by lack of harmonisation and availability of validated serological tests.

Development of novel and next-generation diagnostic technologies for CCHF would benefit from a refined set of target product profiles (TPP) with detailed clinical and operational design specifications, including a range of minimal to optimal performance characteristics. Application-driven TPPs can be designed to support the development of CCHF diagnostics that have been identified here to accelerate care and minimise transmission risk: POC diagnostics for patient triage, screening and field testing; syndromic PCR panels for expediting differential diagnosis of CCHF from other VHF pathogens; and NGS to monitor circulating strains and viral mutations, particularly to assess the sensitivity of probe design used in molecular diagnostics. Ongoing initiatives include a CCHF-specific TPP currently being developed by WHO as part of their roadmap.^112^
