# Supplementary material for: Diagnostic tests for Crimean-Congo haemorrhagic fever: a widespread tickborne disease
Source: BMJ Glob Health. 2019 Feb 20;4(Suppl 2):e001114. doi: 10.1136/bmjgh-2018-001114 (PMC6407549; doi:10.1136/bmjgh-2018-001114)
Supplement: Supplementary data [file bmjgh-2018-001114supp001.pdf]

Table S1: Molecular Diagnostic Tests for CCHF

Commercial and regulated assays for CCHF are presented. Legacy lab-developed tests and in-house assays are not presented here, as sensitivity/specificity/LOD data is lab-specific (see CCHF Matrix of Suppliers.xlsx for further detail).

Developer	System	Regulation	Sample Type	Target	LOD	Sensitivity /PPA	Specificity /NPA	Specimens tested	Reference assay
**Commercial – single assays**									
**Altona Diagnostics** GmbH (GER)	**RealStar® CCHFV RT-PCR Kit**	CE-IVD	Extracted RNA is the starting material for the RealStar® CCHFV RT-PCR Kit 1.0.	CCHFV RNA (target not specified)	14.2 copies/µl<br><br>$1.4x10^4$ cp/mL	no clinical specimens	no clinical specimens	quantified RNA (in vitro transcripts) extracted from different pathogens causing febrile diseases	no info
**Fast-track Diagnostics** (Malta) (partnered with Siemens 2017)	**FTD CCHFV** FTlyo CCHFV (master mix)	CE-IVD	This test is for use with extracted nucleic acid from whole blood of human origin.	CCHFV RNA (target not specified)	1×10^3 copies/mL	86%<br>100% vs competitor	100%	Sens: 43 total samples<br>Spec: 93 clinical samples	In-house ref (Escadafal 2011) and competitor (not specified)
**Bio SB / LifeRiver** (USA)<br>Shanghai ZJ Bio-Tech Co., Ltd (China)	**Crimean-Congo Hemorrhagic Fever Virus (CCHFV) Real Time RT-PCR Kit**	CE-IVD	serum, plasma or insect vector	CCHFV RNA (target not specified)	1×10^3 copies/mL<br>Linear range: 2×10^3 to 1×10^8 copies/ml	no info	no info	no info	no info
**genesig** (UK) (Primerdesign Ltd)<br><br>same kit as **PCRmax** (UK)	**genesig® q16 CCHFV-EASY**	RUO	All kinds of sample material suited for PCR amplification can be used.	highly conserved regions within nucleocapsid gene of the S segment	< 100 copies of target	100% homology with a broad range of CCHFV sequences based on a comprehensive bioinformatics analysis.	no clinical specimens	Synthetic CCHF RNA	no info
**VL Diagnostics/LIPSGene** (GER)<br>dist. **Bioactiva Diagnostica**<br>dist. **BioDiag ME**	**LIPSGENE CCHF Kit**	CE-IVD	no info	CCHFV RNA (target not specified)	≥10 copies/run	no info	no info	Synthetic CCHF RNA	no info

**BioinGentech** (Chile)	**HumPCR-realtime™ CCHF Detection Kit - Real time**	RUO	blood, serum, animal and plant cells and tissue, bacteria and yeast	CCHFV RNA (target not specified)	no info	no info	no info	no info	no info
**BioinGentech** (Chile)	**VetPCR-realtime™ CCHF Detection Kit - Real time**	RUO	blood, serum, animal and plant cells and tissue, bacteria and yeast	CCHFV RNA (target not specified)	no info	no info	no info	no info	no info
**Sacace Biotechnologies** (Italy)	**Congo-Crimea Real-TM**	CE-IVD	plasma, ticks	CCHFV RNA (target not specified)	10^3 copies/ml	no clinical specimens	100%	RNA from West other virus and pathogens	no info
**Ecoli Ltd.** (Slovak Republic)	**AmpliSensÒ CCHFV-FRT PCR kit**	CE-IVD	blood, plasma, serum and ticks	CCHFV RNA (target not specified) - amplification of pathogen genome specific region using specific CCHFV primers	$5x10^3$ copies/mL	no clinical specimens	100%	RNA from West other virus and pathogens	no info
**Commercial – multiplex assays**									
**Developer**	**System**	**Regulatory status**	**Sample Type**	**Target**	**LOD**	**Sensitivity /PPA**	**Specificity /NPA**	**Specimens tested**	**Reference assay**
**Luminex GenArraytion Inc.** (USA)	**xMAP MultiFLEX Febrile Agent Panel 2**	RUO	purified samples	CCGFV (3 targets) includes multiple primers and beads for enhanced coverage	<10 copies per rxn	Panel for 9 agents: CCHF, ebola, Lesihmania, Marburg, RVF...	no info	purified genomic material	Agilent microarray
**GenArraytion Inc.** (USA) for Luminex xMAP or TaqMan	**Mega Febrile Illness MULTIFLEX**	RUO	purified samples	CCGFV (3 targets) includes multiple primers and beads for enhanced coverage	<10 copies per rxn	Panel for 21 agents: CCHF, ebola, Marburg, RVF, Lassa, leishmania, leptospir,..	no info	purified genomic material	Agilent microarray
**GenArraytion Inc.** (USA) for Luminex xMAP or TaqMan	**BioThreat MULTIFLEX**	RUO	purified samples	CCGFV target	<10 copies per rxn	Panel for 26 agents: CCHF, ebola, marburg, lassa, vaccinia, variola...	no info	purified genomic material	Agilent microarray
