# Supplementary material for: Diagnostic tests for Crimean-Congo haemorrhagic fever: a widespread tickborne disease
Source: BMJ Glob Health. 2019 Feb 20;4(Suppl 2):e001114. doi: 10.1136/bmjgh-2018-001114 (PMC6407549; doi:10.1136/bmjgh-2018-001114)
Supplement: Supplementary data [file bmjgh-2018-001114supp003.pdf]

Table S2: Serology Tests for CCHF

Commercial and regulated assays for CCHF are presented. Legacy lab-developed tests and in-house assays are not presented here, as sensitivity/specificity/LOD data is lab-specific (see CCHF Matrix of Suppliers.xlsx for further detail).

Commercial ELISA and IFT/IFA									
**Developer**	**System**	**Regulatory status**	**Sample Type**	**Target**	**LOD**	**Sensitivity /PPA**	**Specificity /NPA**	**Specimens tested**	**Reference assay**
**Alpha Diagnostic International / ADI** (US)	**Crimean-Congo hemorrhagic fever virus (CCHFV) IgG, IgM ELISA Kits**	RUO	human serum or plasma of vaccinated, immunized and/or infected hosts	purified recombinant CCHFV nucleoprotein (NP, 482-aa)	no info	no clinical samples	no info	no info	no info
**EUROIMMUN** (GER) (acquired by Perkin Elmer 2017)	**IIFT and IFA BIOCHIPs** (ELISA in development)	CE-IVD	Serum, plasma, culture media or any other biological fluid	CCHF-IgG, CCHF-IgM two recombinant proteins CCHFV-GPC and CCHFV-N transfected cells	IgG: 1:100<br>IgM: 1:10	IgG: 89.5%<br>IgM: 97.2%	IgG: 100%<br>IgM: 97.5%	IgG: 206 positives; 88 neg<br>IgM: 184 positives, 204 neg	no info
**Vector-Best** (RUS)	**VectoCrimea-CHF-IgG VectoCrimea-CHF-IgM VectoCrimea-CHF-antigen**	no info	no info	no info	no info	no info	no info	no info	no info
**Abbexa** (UK)	**Human CCHF-IgG ELISA Kit**	RUO	serum, plasma, tissue, other biological fluids	96 well plate has been pre-coated with an antigen specific to CCHF-IgM.	no info	no info	no info	no info	no info
**Creative Diagnostics** (USA)	**CCHF-IgM ELISA Kit**	RUO	serum, plasma, culture media or any biological fluid	CCHFV nucleoprotein	no info	no info	no info	no info	no info
**Bernhard Nocht Institute for Tropical Medicine** (GER)	**BLACKBOX CCHFV IgG, IgM ELISA Kits**	RUO	human serum	HRP-labelled CCHFV recombinant antigen	Calibrated index value	no info	no info	no info	no info
