# Supplementary material for: Diagnostic tests for Crimean-Congo haemorrhagic fever: a widespread tickborne disease
Source: BMJ Glob Health. 2019 Feb 20;4(Suppl 2):e001114. doi: 10.1136/bmjgh-2018-001114 (PMC6407549; doi:10.1136/bmjgh-2018-001114)
Supplement: Supplementary data [file bmjgh-2018-001114supp002.pdf]

List S1: International References

Several international reference institutes provide specimens for validation or EQA/proficiency. These groups typically have a defined pathogen/disease focus with a corresponding archive of biological reference materials, and the supplies may be limited.

- The European Network for Diagnostics of Imported Viral Diseases (ENIVD) established an expert laboratory network (EVD-LabNet, www.evd-labnet.eu) to provide expert laboratory support for networking, external quality assessments and training of laboratories involved in (re)emerging viral diseases.
- The National Institute for Biological Standards and Control (NIBSC, www.nibsc.org) provides a range of biological reference materials and reagents, including influenza reagents, Quality Control Reagents Unit reagents and other CE-marked IVD reagents.
- The Paul Ehrlich Institute (PEI, www.pei.de) has developed reference materials for serological testing, nucleic acid amplification techniques, and immunoglobulin preparations; PEI is also a resource for international standards and reference panels.
- INSTAND e. V. (www.instand-ev.de) is an interdisciplinary, not-for-profit, scientific medical society, organising EQAs as part of external quality assurance for nearly all areas of laboratory diagnostics.
- The European Virus Archive (EVAg, www.european-virus-archive.com) is a non-profit organisation with expertise in virology dedicated to the characterization, conservation, production, and distribution of biological materials in the field of virology.
- ATCC (www.atcc.org) is the premier global biological materials resource and standards organization offering an extensive collection of products and services manufactured under ISO certification and accreditation.
- The European Directorate for the Quality of Medicines & HealthCare (EDQM, www.edqm.eu) supplies chemical and biological reference preparations for the tests and assays to be carried out in accordance with the official methods prescribed in the European Pharmacopoeia.
- Quality Control for Molecular Diagnostics (QCMD, www.qcmd.org) is an independent International External Quality Assessment (EQA) / Proficiency Testing (PT) organisation for molecular diagnostics, providing laboratories with samples designed to resemble clinically significant specimens to assess specific analytical assay characteristics.
